# Detection of Malicious Cloud Bandwidth Consumption in Cloud Computing Using Machine Learning Techniques

**DOI:** 10.1155/2022/4003403

**Published:** 2022-09-05

**Authors:** Duggineni Veeraiah, Rajanikanta Mohanty, Shakti Kundu, Dharmesh Dhabliya, Mohit Tiwari, Sajjad Shaukat Jamal, Awal Halifa

**Affiliations:** ^1^Department of CSE, Lakireddy Bali Reddy College of Engineering (Autonomous), Mylavaram 521230, NTR District, Andhra Pradesh, India; ^2^Jawaharlal Nehru Technological University Kakinada, Kakinada, East Godavari, India; ^3^Department of CSE-SP FET, Jain University, Bangalore, Karnataka, India; ^4^Directorate of Online Education, Manipal University Jaipur, Jaipur, Rajasthan, India; ^5^Department of Information Technology, Vishwakarma Institute of Information Technology, Pune, Maharashtra, India; ^6^Department of Computer Science and Engineering, Bharati Vidyapeeth's College of Engineering, Delhi, India; ^7^Department of Mathematics, College of Sciences, King Khalid University, Abha, Saudi Arabia; ^8^Kwame Nkrumah University of Science and Technology, Kumasi, Ghana; ^9^Department of Electrical and Electronics Engineering, Tamale Technical University, Tamale, Ghana

## Abstract

The Internet of Things, sometimes known as IoT, is a relatively new kind of Internet connectivity that connects physical objects to the Internet in a way that was not possible in the past. The Internet of Things is another name for this concept (IoT). The Internet of Things has a larger attack surface as a result of its hyperconnectivity and heterogeneity, both of which are characteristics of the IoT. In addition, since the Internet of Things devices are deployed in managed and uncontrolled contexts, it is conceivable for malicious actors to build new attacks that target these devices. As a result, the Internet of Things (IoT) requires self-protection security systems that are able to autonomously interpret attacks in IoT traffic and efficiently handle the attack scenario by triggering appropriate reactions at a pace that is faster than what is currently available. In order to fulfill this requirement, fog computing must be utilised. This type of computing has the capability of integrating an intelligent self-protection mechanism into the distributed fog nodes. This allows the IoT application to be protected with the least amount of human intervention while also allowing for faster management of attack scenarios. Implementing a self-protection mechanism at malicious fog nodes is the primary objective of this research work. This mechanism should be able to detect and predict known attacks based on predefined attack patterns, as well as predict novel attacks based on no predefined attack patterns, and then choose the most appropriate response to neutralise the identified attack. In the environment of the IoT, a distributed Gaussian process regression is used at fog nodes to anticipate attack patterns that have not been established in the past. This allows for the prediction of new cyberattacks in the environment. It predicts attacks in an uncertain IoT setting at a speedier rate and with greater precision than prior techniques. It is able to effectively anticipate both low-rate and high-rate assaults in a more timely manner within the dispersed fog nodes, which enables it to mount a more accurate defence. In conclusion, a fog computing-based self-protection system is developed to choose the most appropriate reaction using fuzzy logic for detected or anticipated assaults using the suggested detection and prediction mechanisms. This is accomplished by utilising a self-protection system that is based on the development of a self-protection system that utilises the suggested detection and prediction mechanisms. The findings of the experimental investigation indicate that the proposed system identifies threats, lowers bandwidth usage, and thwarts assaults at a rate that is twenty-five percent faster than the cloud-based system implementation.

## 1. Introduction

Attacks of many types, such as distributed denial of service attacks, zero-day assaults, and HTTP attacks, are all capable of jeopardizing the integrity of the security of a network. Several different Intrusion Detection System (IDS) strategies, including host-based IDS, network-based IDS, and signature-based IDS, are used in order to provide adequate defence against attacks of this kind. A distributed denial of service attack, also called a vulnerable assault, has as its main purpose the prevention of users from accessing the resource that is the focus of the attack. Several techniques have been created in order to secure networks against attacks. These strategies include static approaches, knowledge-based strategies, machine learning strategies, and soft computing methods. Because the amount of requests made during a DDoS attack is often significantly more than normal and because the type of DDoS traffic is relatively similar to that of ordinary traffic [1], it is possible to easily identify the attack. The MCBC attack is a novel kind of attack that, in general, disrupts the utility model of online services that are accessible over the Internet. The basis for cloud computing is either the idea of a pay-as-you-go service or the utility pricing model. Cloud computing is a kind of technology that is referred to as “cloud computing.” People are required to make payments for the utilities (such as gas and electricity) that they use on a regular basis in their homes. In a similar vein, users of cloud computing should be compelled to pay for the resources (such as storage and bandwidth) that they make use of [2]. The Distributed Denial of Service Attacks and the Application-Level Distributed Denial of Service Attacks both make use of a vulnerability in the utility model of the cloud, and both have an impact on the cloud consumer application. As a consequence of this, consumers of cloud services have a responsibility to protect their networks by implementing appropriate preventive measures.

## 2. Related Survey

Customers who use cloud services often have concerns about policy-related problems, security, potentially dangerous technical security dangers, and legal challenges. The availability of the CSP is an essential component that plays an important role in determining its viability over the long run. The feature of this component that is of the biggest significance is the constant supply of cloud services. The many attacks, including DOS and DDoS, are having a detrimental effect on the availability of the network. The DDoS attack may be extended into something called an EDoS attack, which stands for extended distributed denial of service. An ED that is easily identifiable, functioning like DDoS [3]. Even though distributed denial of service (EDoS) attacks are becoming more prevalent as cloud computing becomes more widespread, and even though they have a significant impact on distributed denial of service (DDoS) attacks, a growing number of protection and mitigation strategies are being developed in order to foil these kinds of assaults.

The DDoS attack is the most dangerous and pervasive kind of cybercrime that can be found on the Internet. An application-layer-based DDoS attack masquerades its intended victims by impersonating them with valid HTTP requests [4]. It is possible that the rate of web requests may increase significantly to a great degree as a result of the concentration of a big number of standard consumers on certain content (known as the flash crowd). When the unexpectedly large number of individuals arrived all at once, the server would have trouble keeping up with the very high volume of work being completed. This presents a substantial challenge for attacking the flash in terms of quickly recognising the DDoS attack.

## 3. Malicious Cloud Bandwidth Consumption Attack

**Figure 1:**
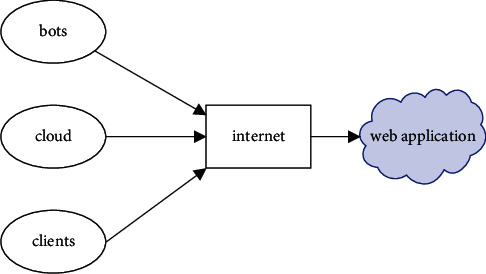
MCBC attack scenario.

**Figure 2:**
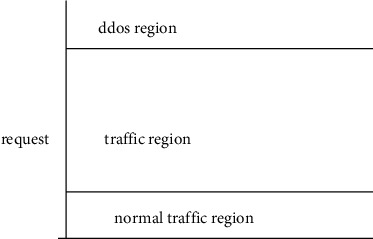
MCBC attack region.

The primary objective of the attacker in this kind of assault is to make repeated use of the resources in such a manner that the availability of those resources is not adversely affected but that the utility model is gradually impacted instead. The MCBC assault, in contrast to the DDoS attack, is carried out over a prolonged length of time. This allows the attacker to gain over a longer period while the customer suffers a financial loss as a result of the attack. An attacker and a typical user are required components of the MCBC attack scenario. These forms of harmful assaults do not have an effect on the user in the form of the consumption of resources; rather, the user experiences a loss of money value [5]. The MCBC assault scenario and the areas are shown in Figures 1 and 2, respectively.


(1) $$\begin{array}{c} r_{t}=AWb_{i}+v_{t},\\ d_{i}=y_{i}=S^{T}r_{i},\\ SF=\frac{256}{2^{k}}. \end{array}$$

### 3.1. The Threat Model of the MCBC

**Figure 3:**
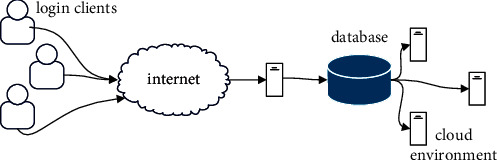
MCBC threat model.

Figure 3 provides an overview of an EDoS assault scenario and its players. The participants in an EDoS scenario include CSPs, victim servers, legitimate clients, and malicious clients (i.e., attackers).

(2) $$\begin{array}{c} Y\left(k\right)=\frac{1}{N_{A}}\sum_{n=1}^{N_{A}}{\left|h_{k}\left(n\right)\right|}^{2}\\ =\tilde{p}\left(k\right)+{\tilde{\sigma }}_{I}^{2}\left(k\right),\\ {\hat{\sigma }}_{I}^{2}\left(k\right)=\frac{1}{N_{A}}\sum_{n=1}^{N_{A}}{\left|h_{k}\left(n\right)-\sqrt{p\left(k\right)}\alpha_{1k}\left(n\right)\right|}^{2},\\ Z\left(k\right)=Y\left(k\right)-{\hat{\sigma }}_{I}^{2}\left(k\right),\\ D^{2}\left(x,y\right)=\sum_{x=0}^{m}\sum_{y^{\prime }=0}^{n}{\left[f\left(x^{\prime },y^{\prime }\right)-t\left(x^{\prime }-x,y^{\prime }-y\right)\right]}^{2}, \end{array}$$

where *t*(*x*, *y*)is the template and M, N is the size of the template.

(3) $$\begin{array}{c} \sum_{x{\;}^{\prime }=1}^{M}{\sum_{y{\;}^{\prime }=1}^{N}\left[f\left(x{\;}^{\prime },y{\;}^{\prime }\right)-t\left(x{\;}^{\prime }-x,y{\;}^{\prime }-y\right)\right]}^{2}=2\sum_{x{\;}^{\prime }=1}^{M}\sum_{y{\;}^{\prime }=1}^{N}f\left(x{\;}^{\prime },y{\;}^{\prime }\right)t\left(x{\;}^{\prime }-x{\;}^{\prime }y{\;}^{\prime }-y\right), \end{array}$$

where ∑_*x*′=1_^*M*^∑_*y*′=1_^*N*^*f*(*x*′, *y*′)^2^+ ← Background, ∑_*x*=1_^*M*^∑_*y*=1_^*N*^*t*(*x*′ − *x*, *y*′ − *y*)^2^− Constant, and correlation: convolution of *f*(*x*, *y*) with *t*(−*x*, −*y*).

Legitimate clients: genuine customers are everyday people who take advantage of the offered services; in the end, it is these individuals that generate revenue for the businesses that cater to hosted clients. The word “bot” refers to a script that can carry out a certain function and repeat that work more rapidly than a human being could. The term “botnet” refers to what happens when a large number of bots are distributed over several computers, all of which are then connected to one another through the Internet. A botnet is an abbreviation that stands for “network of bots” and refers to the collection of automated software programs that are referred to collectively as a botnet. When referring to the individual who is in charge of the command and control of a botnet that is used for remote process execution, the term “botmaster” is employed [6]. The botmaster is the one that is accountable for commanding the whole of the botnet via the use of the Internet and the command and control server, as well as delivering orders to all of the bots that are operating on the compromised targets [7]. It is the responsibility of the botmaster to determine whether or not detrimental actions have been taken and to monitor the targets that have been affected [8].

### 3.2. Examples of Regular and MCBC Traffic Patterns

**Figure 4:**
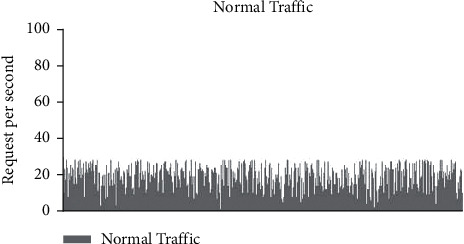
Normal behaviour graph.

**Figure 5:**
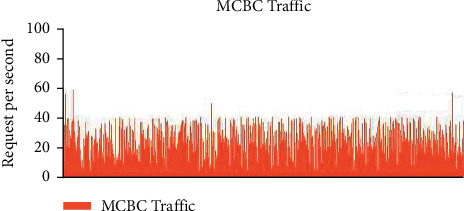
MCBC behaviour graph.

The typical traffic pattern of the dataset indicates that user queries fall within the range of between 5 and 10 requests and do not exceed more than 20 requests. This was discovered by observing that the maximum number of requests that may be made is 20. The range in question does not go beyond the range.  Figure 4 depicts the normal flow of traffic, and upon closer analysis of the image, it is obvious that the request pattern is well within the acceptable range of 20 requests [9]. This is shown by the fact that the flow of traffic is depicted in the figure.  Figure 5 illustrates the malicious traffic that results in requests that use up a lot of resources [10]. These requests are not inside the DDoS zone, but they are much higher than the average range [11].  Figure 4 presents a graphical representation of the combined MCBC activity and normal activity that takes place [12]. The graph clearly demonstrates that the MCBC traffic is operating in the same way as the normal traffic and using resources with the intention of creating a financial burden on the consumer.  Figure 5 shows the average monthly client requests for one month [13].

**Figure 6:**
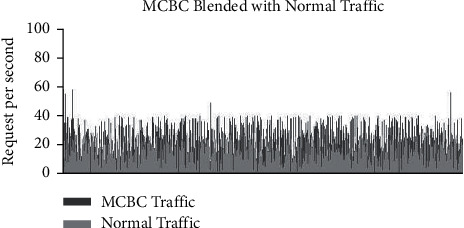
MCBC blended with normal traffic.

**Figure 7:**
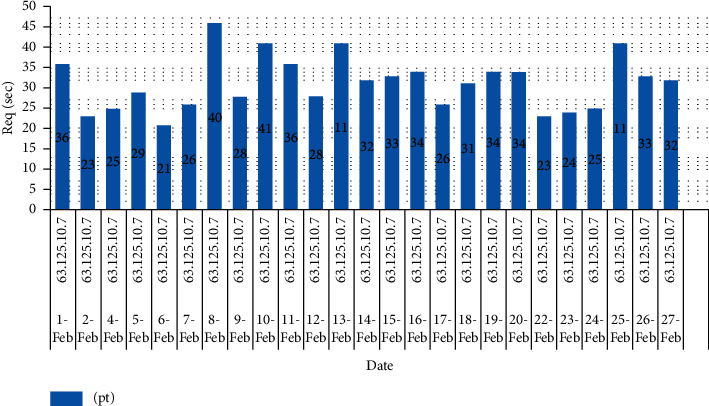
Per client request volume that falls in MCBC region for 1 month.

Figures 6 and 7 represent the overall survey for 1 month of the proposed work.

## 4. Web Server Access Log Format and Description

Web servers' log consists of user activity in the form of requests, which are processed by the server. The log files in various web servers sustain various kinds of information [14]. The log format directive is employed to simplify the selection of the contents of the logs. The basic configuration in the Common Log Format (CLF) file is shown in Table 1.

### 4.1. Web Log Mining: Data Preprocessing

**Figure 8:**
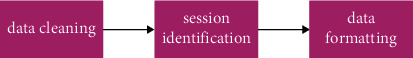
General Stages of data preprocessing.

**Figure 9:**
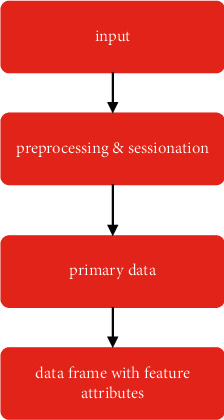
Stages and result of data preprocessing.

Blog mining is the only kind of programme that can establish user access patterns from web server logs, making it an essential part of the data mining methodology. The online use statistics collect information about how people navigate the Internet using a certain website. The log files include a variety of information, including the number of bytes transmitted [15], the user's IP address, the user's user name, the time stamp, the access request, the result status, and the user agent. All of the different sorts of requests that are dealt with by the server are logged in the server access log [16]. It is not feasible to immediately utilise the data obtained from online access logs in the process of pattern search and mining. After using the data in the pattern identification process, it is necessary to preprocess the data. The preparation procedures, together with the final dataset performance, are shown in Figures 8 and 9. The raw access logs cannot be directly utilised for mining the MCBC attacks because the necessary variables are not included in the raw log. As a result, the logs need to be preprocessed in order to create the necessary variables, which are mentioned in Table 2.

Before the genuine logs can be evaluated, the unwanted requests that are included in the raw logs need to be removed from them. If the requests' status codes are anything other than 2XX, we do not want to receive them and consider them to be unwanted. Requests that have a status code that is anything other than 2XX do not contribute to the MCBC attack or, to put it another way, make use of the bandwidth [17]. The requests have been compiled into a list, which can be seen in Table 1, along with an explanation of each one. As a consequence of this, the process of data preparation plays an important role in evaluating whether or not an MCBC attack has taken place.  Figure 8 illustrates the processes that are involved in the preprocessing stage of the method. It is vital to clean the data, which involves getting rid of queries that are not required and do not contribute to the bandwidth utilisation attack. The last stage is to identify sessions for each host that is sending requests. This is required since sessions are one of the variables that are utilised in MCBC detection; thus, identifying sessions is the next step. In the last phase, the data will be formatted into a data frame that the machine learning algorithm will be able to recognise. This will allow the programme to evaluate and identify potential risks.  Figure 9, which may be seen below [18], illustrates the consequences that these activities have on the environment.

## 5. Data Cleaning

During the process of data cleaning, redundant and unnecessary data are extracted from the web server logs and deleted from the system. Requests with status codes other than 2xx are taken into consideration for cleaning since they do not contribute to the use of the available bandwidth. The log file contains the following three categories of data that are unnecessary, redundant, and irrelevant.

The intended websites are typically recorded in several log file entries when they are incorporated into an HTML file. After the core requests for web pages, the embedded graphical requests with an extension such as gif, jpeg, GIF, JPEG, jpg, JPG, CSS, or CGI come next. Simply keeping the core needs in mind results in the elimination of these subsidiary requests.

**Robots requests**: Numerous search engines make use of online robots known as spiders on occasion. These web robots will supply an accurate method for conducting searches on websites and will update the search weblog file of various sorts of automated access activity.

**Error request**: A file with erroneous information is insufficient for WUM, and the HTTP status codes have been eliminated. For example, if the entry is deleted and the status code is 404, it indicates that the resource being accessed does not exist and removes the record. In the event that the log entries with a status code between 200 and 299 deliver the successful answer, entries from the log that have other status codes will be erased.

Cleaning up an algorithm: the data.

Input: File Used for Tracking Web Access.
As a result, the Web Access Log File *L* has been cleaned.
(1) Concerning every AccessLogRecord included inside the Web Access Log file
(2) if the condition is met, (AccessLogRecord request does not equal ∗.gif | ∗.jpeg | ∗.jpg | ∗.css), then continue.
(3) AND (the GET technique was used to access the log record).
(4) AND (AccessLogRecord status is the same as 200)
(5) If the AccessLogRecord user agent does not match any of the following: crawler, spider, or robot
(6) Copy the AccessLogRecord into the *L* file.
  1. continue
  2. End For

### 5.1. User Identification

This step identifies unique users based on the new IP address and/or OS browsing software.

### 5.2. Session Identification

A session means a set of web pages visited by a specific client. The methods used to identify user sessions are based on the timeout mechanism and maximal forward reference with the following rules applied to identify a session:

- If a new IP address is registered in a weblog file, a new user and also a new session shall be created.
- If the referrer page in an entry of a web log file is null, then a new session is created.
- If the idle time between page requests exceeds 30 minutes, then the user is assumed to start a new session.

### 5.3. Dataset Description

The other dataset is from honeynet, which is used in the experimental analysis. Also, set up a test web application on IndianVajarahost with the domain name joyhoy.com and collected logs for the experiment. During the preprocessing stage as represented by Figure 8, the weblog was processed to separate the primary requests from the secondary requests. All ∗.html, ∗.html, and the documents requested were considered primary requests. The one with graphics having ∗.css, ∗.jpg, and ∗.jpeg were tagged as secondary requests as discussed in the data cleaning algorithm. The weblog properties that are used in the experiment are shown in Table 2.

Apart from the honeynet dataset for considering requests per second attribute, two other datasets that were used are mentioned in the table. The NASA dataset is the popular research dataset publicly available from a busy NASA web server. The second dataset joyhoy dataset is from the test web application hosted on the Indian Variant host in Table 3.

### 5.4. Algorithm for Traffic Generation

The aim of the proposed traffic generation algorithm is to simulate HTTP web request tra *c* as observed from the actual access log entries is shown in algorithm 1.

This algorithm is a combination of modeling WUM and the learned browsing pattern. So, it focuses on generating individual requests that compose a web session based on learned browsing patterns. The major two components of algorithms are described as follows.

### 5.5. Session Reconstruction

**Figure 10:**
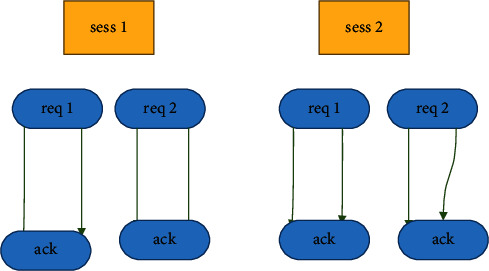
Sessionization steps.

A web application is visited for different purposes. It is possible for a website to be visited by a regular user as a normal (natural) visit and for indexing to be viewed by crawlers, bots, spiders, etc. The proposed session identification technique is a simple time heuristic approach to preprocess the webserver access logs with 30 minutes as a timeout period for a session which is consistent is shown in Figure 10.

The procedure for measuring the arrival time of the session and the following interarrival time of the Request is shown in Figure 10. A session is considered to be active from the moment the initial HTML request is sent until the timeout duration of thirty minutes is reached. Interarrival of requests refers to the arrival of requests one after another within an active session. This continues until either the session is terminated or a new one is initiated.

### 5.6. Recognising the Most Popular Web Requests

Most of the time, a single user request for an HTML page will be followed by a cascade of secondary inline requests to obtain embedded objects like photographs, scripts, and videos. This is because HTML pages are designed to support several types of embedded content. The dimensions of the HTML page itself are mentioned in the weblog item corresponding to this initial request. The follow-up postings in the weblog will be the secondary inline requests that are submitted. This principal web request is singled out by the preprocessing module, which also compares the total size of the secondary requests to that of the primary requests. These secondary requests are not included in the weblog since removing them would make their processing of them easier. Therefore, the weblog only includes entries that pertain to main requests together with the total data amount expressed in bytes.

### 5.7. Detailed Explanations of Each Algorithm

The solution that is housed in the cloud is a compilation of different online documents and services that are made accessible to the general public through the Internet. When customers request a hosted document via their browser, Cloud Servers provide it to them. These hosted documents or pages may include several embedded items, such as texts, photos, scripts, and audio and video files. The majority of the previous research has been conducted in the field of WUM, focusing on topics such as workload characterization [19]. The workload categorization takes into account the document type, size, referencing qualities, and geographic distribution of server requests for the whole of the datasets. Additionally, much more study work on WUM is carried out concerning modeling of the person is overloaded, the response time for web page becomes high, which affects the business or firm. If a visitor waits a long time for a response, they are more likely to pick one of the many other options available on the web; specifically, they will go to a different webshop. The log files are kept on most web servers, and these log files supply the information that is used to generate the workload model. The Hypertext Transfer Protocol (HTTP), which is an application protocol used by both WWW clients and servers, is responsible for the vast majority of the work done on the worldwide Internet. Using a stochastic approach, similar work of producing synthetic HTTP traffic has been discovered [20]. The stochastic model is distinct from the one that we have developed since its primary focus is on the generation of link traffic, while our purpose is to produce an aggregate number of user requests for a particular website. The log repositories play a significant part in the operation of web servers since they are responsible for recording the behaviour of users and supplying a wealth of information about their surfing habits. The process of obtaining the browsing habits of users of a website is referred to as the Web Usage Pattern, and it is accomplished by analyzing the navigational features. The average amount of time that a person spends browsing a website is one of the factors that may be used to identify a web user session. The computation of time intervals of page visits and examinations of those time intervals to get a certain threshold is then used to divide the blogs up into sessions [21].

### 5.8. Production of Traffic Used in Attacks

The dataset that is being used must include the attack traffic of MCBC for there to be the detection of an MCBC attack in the dataset. Both the NASA dataset and the joyhoy dataset, which are both historical, were weaved together using two distinct MCBC assault patterns [22]. The first type of attack is a naïve random or brute force attack, in which the attacker is assumed to not know the website user behaviour. The second type of attacker is a more intelligent one, in which the attacker has a fair knowledge of the top pages or documents of the website that is being attacked [23].

### 5.9. Unpredictable Assault

The arbitrary assault and the prudent attack are both components in the creation of the MCBC attack. Because the attacker has very little prior information about the website being targeted, the arbitrary assault is the sort of attack that is considered to be the most naïve [24]. That is to say, the ignorant attacker is uninformed of the frequency with which websites are accessed as well as the logical context of the pages that are being requested. The attacker will first make seemingly random requests for the websites. The goal of the attacker is to simply use up all of the available bandwidth by requesting web pages in completely random order. The preceding method, which was covered in Section 5, is used to mimic this form of assault on the system. The purpose of this particular kind of attack is to simply make a random request for the number of pages to use up the available resource [25].

### 5.10. An Assault Using a Session Replay

In this kind of attack, the currently active popular sessions are repeated. This type of attack is also known as a prescribed session attack, and it operates on the presumption that the attacker has a good working knowledge of the most popular websites [26]. The attacker makes requests to consume resources without providing any economic value for the host by taking advantage of the fact that they are aware of the behaviour of traffic patterns of the website that is now under attack. For introducing this form of traffic in Log *L*, the traffic is produced using the method mentioned above [27]. This traffic is created by simply replaying or starting to request the website's most popular pages to consume the bandwidth. This form of assault is produced by repeatedly playing the sessions that have had the most requests for the most popular sites.

### 5.11. Restrictions Placed on the Traffic-Generating Algorithm

The actual web pages that users request are what constitutes the training data that is utilised in the web server log entries. The success of artificial log generation methods is highly dependent on the accuracy of the empirical data that are observed [28]. In conclusion, just as we are aware that obtaining genuine data in the security area is usually difficult; the same constraint applies to the current study since there are relatively few live datasets that are readily accessible to the public. In spite of the fact that the current study has this constraint, it is not immediately obvious that the method that has been provided does not match the general solution. Once the performance of the proposed algorithm has been validated against a larger collection of real websites, only then will it be possible to demonstrate the method's generalizability [29].

## 6. Experimental Results

When it came to training and testing for threat detection, the datasets were leveraged to their best potential in every possible way. Logs should include attack traffic if they are going to be useful for detecting MCBC threats in and of themselves [30]. The synthetic traffic pattern was produced by using the algorithm [31]. The sample of two weeks' worth of traffic was taken from the produced traffic to assess whether or not the simulated traffic followed the pattern of real traffic. We employed a graphical way to display the traffic to determine whether or not the pattern of traffic that was created was accurate. To check that the traffic produced and the real traffic had the same statistical behaviour, we conducted the Wilcoxon statistical test [32].

In order to determine whether the generated traffic pattern is valid concerning the null hypothesis, we compared the training data to the test data using a significance level of 0.05 and a confidence interval of 95%. This was done based on the hypothesis that weeks of hosted web traffic shall produce statistically similar distributions [33].

More generally, if we have samples of observations from each of the two populations A and B containing nA and nB observations, respectively [34]. We test the hypothesis that the distribution of X-measurements in population A is the same as that in B, which we will write symbolically as H0: *A* = *B*.

We accept the null hypothesis and conclude that actual traffic to simulated traffic is statistically similar. Also, with the prior knowledge of actual data, logs allowed us to compare the simulated traffic with that of actual traffic [35].

## 7. Conclusion

When it comes to making efficient use of resources, the cloud presents its users with several challenges that must be surmounted. The modeling process should be able to correctly identify the use patterns of the hosted web application in order to facilitate the discovery of a workable solution to the problem. In a nutshell, the following goals were meant to be accomplished with the help of the strategy that was detailed in this chapter: modeling the cost-utility of traffic bandwidth; employing methods for data preparation to classify the page sizes of main and secondary requests; creating a program that will create fake traffic to your blog for a certain amount of days using an algorithm; generating random attack traffic in addition to the session assault traffic that is required; then weaving that traffic into the actual record of online access.

## Figures and Tables

**Algorithm 1:**
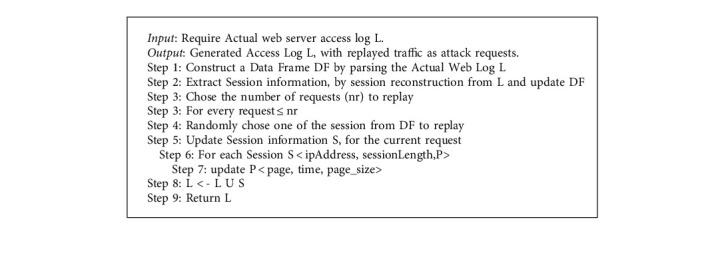
Generate Artificial Log from the Actual Observed Log L.

**Table 1:** Description of the log entry.

Field	Description
199.120.110.21	Client ip address
%*l*	Requested information
%*u*	User id
%*t*	Time format
Get images	Request for logo protocol
%b	Status code

**Table 2:** Weblog properties.

Metric	Description
Number of primary requests	Total number of HTML|HTM requests in the entire data set
Request volume per client	Number of primary requests sent by a client
Request per session	Number of requests per session per client
Session volume	Number of sessions per client
Think time	The time between the completion of one request and the start of the next request
Class	Normal/Malicious (0/1)

**Table 3:** NASA and joyhoy dataset statistical summary.

Duration	Metric	NASA	Joyhoy
4—weeks	Total requests	1891714	327084
	Duration	28 days	28 days

## Data Availability

The data that support the findings of this study are available on request from the corresponding author.

## References

[1] Ambrosin M., Conti M., De Gaspari F., Poovendran R. Lineswitch: Efficiently Managing Switch Flow in Software-Defined Networking while Effectively Tackling Dos Attacks.

[2] Amokrane A., Langar R., Boutaba R., Pujolle G. (2015). Flow-based management for energy-efficient campus networks. *IEEE Transactions on Network and Service Management*.

[3] Jain P., Alsanie W. F., Gago D. O. (2022). A Cloud-Based Machine Learning Approach to Reduce Noise in ECG Arrhythmias for Smart Healthcare Services. *Computational Intelligence and Neuroscience*.

[4] Ashraf J. S. Handling Intrusion and DDoS Attacks in Software-Defined Networks Using Machine Learning Techniques.

[5] Motwani A., Shukla P. K., Pawar M. (2021). Novel framework based on deep learning and cloud analytics for smart patient monitoring and recommendation (SPMR). *Journal of Ambient Intelligence and Humanized Computing*.

[6] Chen Y., Pei J., Li D. DETPro: a high-efficiency and LowLatency system Against DDoS attacks in sdn based on decision tree.

[7] Kushwaha S. S., Joshi S., Singh D., Kaur M., Lee H.-N. (2022). Systematic review of security vulnerabilities in ethereum blockchain smart contract. *IEEE Access*.

[8] Kaur M., Singh D., Kumar V., Gupta B. B., Abd El-Latif A. A. (Sept. 2021). Secure and energy efficient-based E-health care framework for green internet of Things. *IEEE Transactions on Green Communications and Networking*.

[9] Ayachi M. A., Bidan C., Abbes T., Bouhoula A. Misbehavior Detection Using Implicit Trust Relations in the AODV Routing Protocol.

[10] Patil S. D., Raut R., Jhaveri R. H., Dhade P. V., Kathole A. B., Vhatkar K. N. (2022). Robust Authentication System with Privacy Preservation of Biometrics. *Security and Communication Networks*.

[11] Xia W., Neware R., Kumar S. D., Karras D. A., Rizwan A. (2022). An optimization technique for intrusion detection of industrial control network vulnerabilities based on BP neural network. *International Journal of System Assurance Engineering and Management*.

[12] Kumar S., Srivastava P. K., Pal A. K. (2022). Protecting location privacy in cloud services. *Journal of Discrete Mathematical Sciences and Cryptography*.

[13] Rutvij J., Piyush S., Saad A., Deepsubhra G. R., Poongodi M, Malathy S. Smart Tree Health Assessment (THA) model using advanced computer vision techniques and machine learning. *Australia Innovation Patent, Application: 2021104683*.

[14] Barki L., Shidling A., Meti N., Narayan D. G., Mulla M. M. (2016). Detection of distributed denial of service attacks in software-defined networks. *International Conference on Advances in Computing, Communications, and Informatics*.

[15] Bawany N. Z., Shamsi J. A., Salah K. (2017). DDoS attack detection and mitigation using SDN: methods, practices, and solutions. *Arabian Journal for Science and Engineering*.

[16] Stalin S., Maheshwary P., Shukla P. K., Tiwari A., Khare A., Singh U. P., Tiwari A. (2018). Fast chaotic encryption using circuits for mobile and cloud computing: investigations under the umbrella of cryptography. *Soft-Computing-Based Nonlinear Control Systems Design, Rajeev Kumar Singh*.

[17] Kaur M., Singh D. (2021). Multiobjective evolutionary optimization techniques based hyperchaotic map and their applications in image encryption. *Multidimensional Systems and Signal Processing*.

[18] Behal S., Kumar K., Sachdeva M. (2018). D-FACE: an anomaly-based distributed approach for early detection of DDoS attacks and flash events. *Journal of Network and Computer Applications*.

[19] Beheshti N. Y. (2012). Fast failover for control traffic in software-defined networks. *Global Communications Conference*.

[20] Berman M., Chase J. S., Landweber L. (2014). GENI: a federated testbed for innovative network experiments. *Computer Networks*.

[21] Gupta R. K., Almuzaini K. K., Pateriya R. K., Shah K., Shukla P. K., Akwafo R. (2022). An improved secure key generation using enhanced identity-based encryption for cloud computing in large-scale 5G. *Wireless Communications and Mobile Computing*.

[22] Boite J., Nardin P. A., Rebecchi F., Bouet M., Conan V. StateSec: Stateful Monitoring for DDoS protection in Software-Defined Networks.

[23] Rodrigo B., Mota E., Passito A. Lightweight DDoS Flooding Attack Detection Using NOX/open Flow’, Local Computer Networks.

[24] Brooks M. B. A man in the middle attack against open daylight SDN controller.

[25] Patel C., Ali K., Ahmad A. A., Rutvij H. J. (2022). EBAKE-SE: a novel ecc based authenticated key exchange between industrial IoT devices using secure element. *Digital Communications and Networks*.

[26] Parashar V., Kashyap R., Rizwan A. ., R. (2022). Aggregation-based dynamic channel bonding to maximise the performance of wireless local area networks (WLAN). *Wireless Communications and Mobile Computing*.

[27] Pandey A., Shukla P. K., Agrawal R. (2022). Salp swarm optimization-based clustering algorithm (SSOCA) in adaptive FANET to improve QoS for disaster response operations. *Wireless Personal Communications*.

[28] Kumar S., Dubey K. K., Gautam A. K., Verma S., Kumar V., Mamodiya U. (2022). Detection of recurring vulnerabilities in computing services. *Journal of Discrete Mathematical Sciences and Cryptography*.

[29] Sathya M., Jeyaselvi M., Krishnasamy L., Hazzazi M. M. (2021). Prashant kumar shukla, piyush kumar shukla, stephen jeswinde nuagah, A novel, efficient, and secure anomaly detection technique using DWU-ODBN for IoT-enabled multimedia communication systems. *Wireless Communications and Mobile Computing*.

[30] Callegati F, Cerroni W, Ramilli M (2009). Man-in-the-middle attack to the HTTPS protocol. *IEEE Security and Privacy*.

[31] Ahanger T. A., Aljumah A., Atiquzzaman M. (2022). State-of-the-art survey of artificial intelligent techniques for IoT security. *Computer Networks*.

[32] Chen K. Y., Junuthula A. R., Siddhrau I. K., Xu Y., Chao SDN Shield: Towards More Comprehensive Defense against DDoS Attacks on SDN Control Plane.

[33] Jhaveri R. H., Patel N. M. (2017). Attack-pattern discovery based enhanced trust model for secure routing in mobile ad-hoc networks. *International Journal of Communication Systems*.

[34] Chen P. J., Chen Y. W. Implementation of SDN Based Network Intrusion Detection and Prevention System.

[35] Pareek P. K., Sridhar C., Kalidoss R. (2022). IntOPMICM: intelligent medical image size reduction model. *Journal of Healthcare Engineering*.

